# P-264. Fournier-transform Infrared spectrometry is a quick, reliable and economical method used to determine clonality and nosocomial transmission of an extended Spectrum β-lactamase *E.coli* contaminated endoscope

**DOI:** 10.1093/ofid/ofae631.468

**Published:** 2025-01-29

**Authors:** Stephen A De Jager, Carolyn Moonen, Marit Visser

**Affiliations:** Comicro/ Dijklander Hospital, HOORN, Noord-Holland, Netherlands; Bruker Daltonics GmbH & Co. KG, Bremen, Bremen, Germany; Comicro, HOORN, Noord-Holland, Netherlands

## Abstract

**Background:**

Nosocomial transmission of bacteria via contaminated endoscopes is known to occur due to inadequacies in the complex cleaning process of multi-channel endoscopes. At our institution we do regular surveillance culture screening of endoscopes. Upon receiving positive cultures with an Extended Spectrum β-lactamase producing (ESBL) *E.coli* we alerted our hospital hygiene unit. After screening potentially exposed patients we determined clonality of ESBL *E.coli* isolates from 4 patients with expensive en specialized molecular diagnostics, at our local academic hospital using Amplification Fragment Length Polymorphism (AFLP). We were testing the BiotyperIR (Bruker Daltronics) in our laboratory and wanted to see how de FTIR compared to AFLP in our small peripheral hospital setting.

BiotyperIR 3D scatterplot

**Figure:**
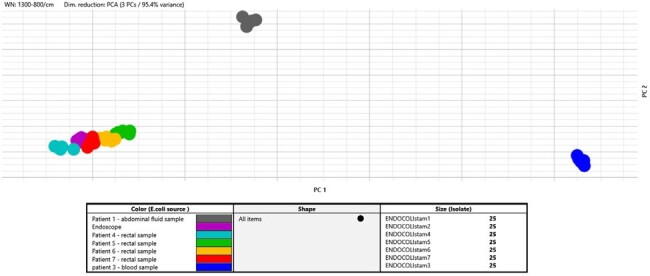

Cluster of endoscope isolate and exposed patient isolates with 2 non related patient control isolates

**Methods:**

We determined that a total of 16 patients had endoscopic investigations preformed with the contaminated endoscope. We were able to do rectal screening cultures for ESBL positive organisms using ESBL selective media from 11 of the 16 patients. We were unable to screen 5 patients. Of the 11 patients that were screened 4 were positive with an ESBL *E.coli*. Clonal testing was done on these isolates and 2 non outbreak ESBL *E.coli* patient isolates with AFLP and FTIR using the BiotyperIR. IR spectra were acquired from dried spots of bacterial suspensions in ethanol solution. Exploratory data analysis of the FTIR spectra was performed by Hierarchical Cluster Analysis (HCA) using Bruker’s updated software.

BiotyperIR dendrogram

**Figure:**
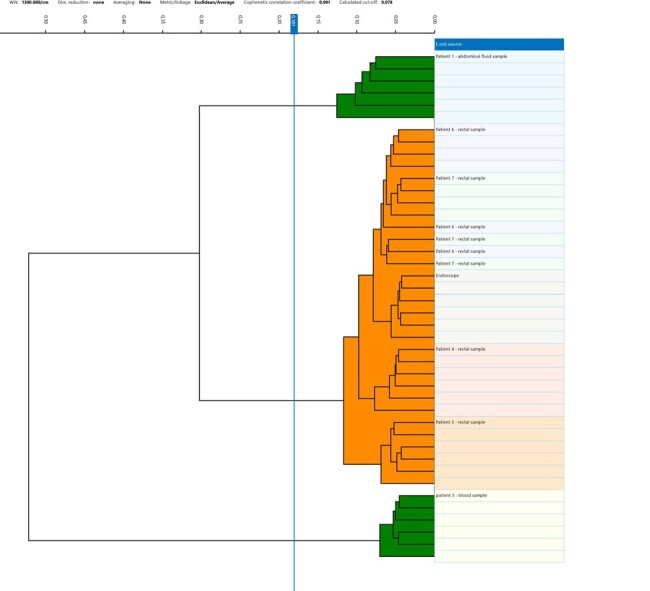

Cluster of endoscope isolate and exposed patient isolates with 2 non related patient control isolates

**Results:**

Both results from AFLP and the BiotyperIR showed that all 4 ESBL *E.coli* patient isolates were identical to the contaminated endoscope isolate and the 2 non outbreak patient control isolates were unrelated to the outbreak isolate. We concluded that there had been nosocomial transmission of an ESBL *E.coli*. Clonal typing using the BiotyperIR FTIR was done within 24 hours in our own laboratory compared to 2 weeks using AFLP which had to be sent to our local academic hospital.

**Conclusion:**

The BiotyperIR FTIR spectrometry results were identical to molecular typing using AFLP to determine nosocomial transmission of a clonal ESBL E.*coli*. Results were obtained significantly faster and at a fraction of the cost of molecular AFLP.

**Disclosures:**

**Carolyn Moonen, PhD**, Bruker Daltonics GmbH & Co. KG: Advisor/Consultant

